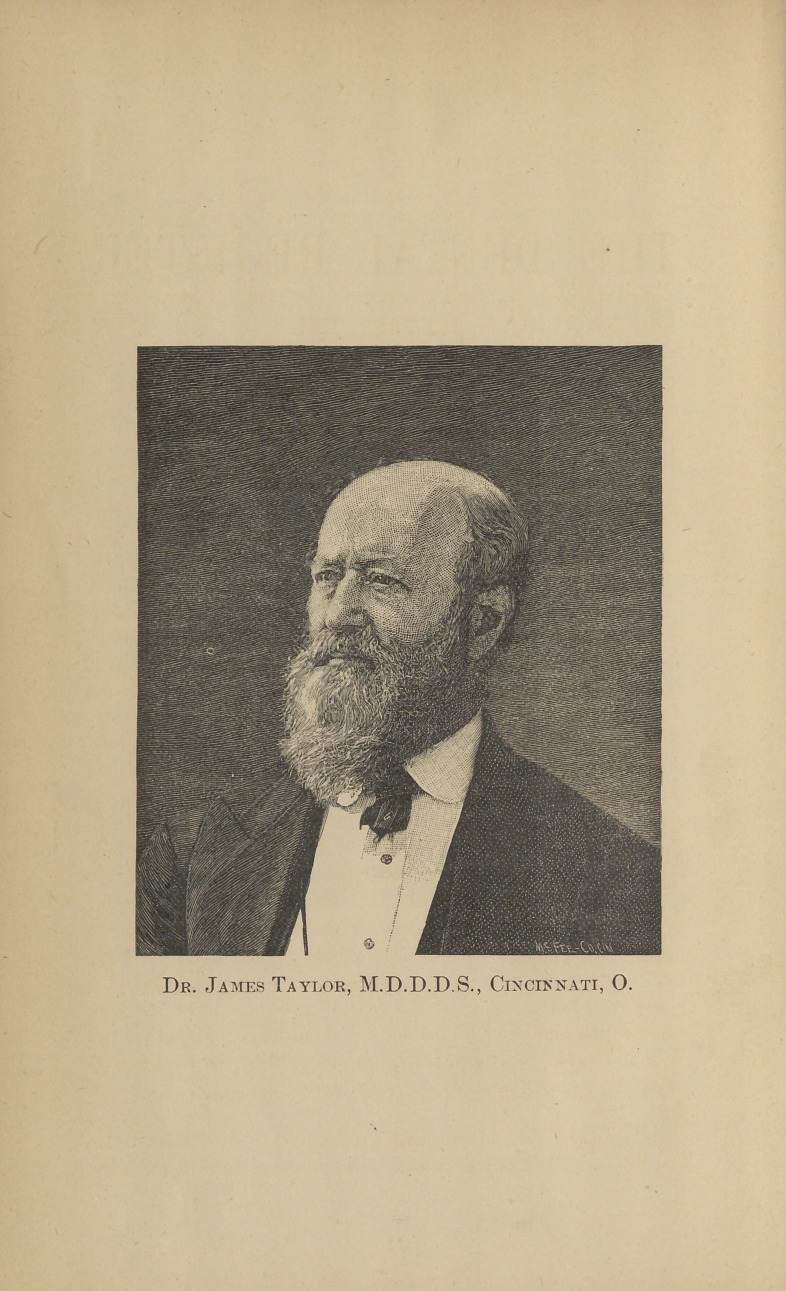# Biographical

**Published:** 1884-01

**Authors:** 


Dr. James Taylor, M.D.D.D.S., Cincinnati, O.

3375-37

### THE DENTAL REGISTER.

VOL. XXXVIII.] JANUARY, 1884. [No. 1.

### BIOGRAPHICAL.

### Biographical.

DR. JAMES TAYLOR, M. D. D. D. S., CINCINNATI, OHIO.

Dr. James Taylor, M.D., D.D.S., was born at Cedar Grove Farm, Paint Creek, near Bainbridge, Ross Co., Ohio, in 1809. The town of Bainbridge was named for Commodore Bainbridge, of the United States Navy, by the grandfather of the subject of this memoir, who was a near relative of the commodore. The old farm is still owned by Price Taylor, a brother of James. Joseph Taylor, the father of this sketch, was born in Monmouth County, New Jersey, where the Taylor family, which was of English descent, settled two hundred years ago, and his mother, whose maiden name was Jane Irwin, was born in Virginia, and was married to Joseph Taylor, in 1797. In 1801, the young pair, with the husband's father, William Taylor, moved to Ross County, being among the first settlers there. Here James Taylor was reared, contending with great obstacles in securing a limited education, by reason of the limited resources of the country. The father, being Magistrate and School Commissioner, employed New England teachers, who were often graduates of colleges, and made his house their home. A good English education was in this way secured to the children. At the age of seventeen James had chosen medicine as his future profession, and advised by the family physician he began the study of Latin and Anatomy at the same time.

In 1826, Dr. John Harris settled in Bainbridge—a man of excellent reputation as a physcian, and, of the students who sought his instruction, young Taylor was soon numbered. Dr. Harris soon after this turned his special attention to the study and practice of dentistry. The works of Bell, Fitch, and Kœcker were procured and eagerly read by the doctor and his student. After a time the latter was sent to Cincinnati to purchase the requisite material for work. The trip was made on horseback, and several days were employed in obtaining files, scalors, forceps, elevators, turnkeys, hippopotamus tusks, gold, tin foil, etc. A set of instruments worth fifty dollars were not then to be obtained in the city. On his return home he found his horse lamed at Batavia, and to occupy the time of his delay, he began to practice with such success that he soon made enough money to pay for his new instruments and the whole expenses of the trip. Some of these first patients gave him their practice when in after years he had settled in the Queen City. The teacher and pupil, now being in partnership, visited various neighboring towns, among them Greenfield, twelve miles distant, where Dr. Chapin A. Harris, a brother of Dr. John, was then practicing medicine. Dr. C. A. Harris, afterward of Baltimore, was soon induced to devote himself also to dental science, and with his industry, integrity, and professional pride, proved a great acquisition to the profession. After two years Dr. John Harris removed permanently to Chillicothe, and young Taylor to Hillsboro, where he placed himself under the instruction of Dr. Kirby, a noted and eminent physician of that town. His dental practice increased to such an extent that it interfered with his medical studies, and he decided to enter the medical school of Transylvania University, Lexington, Kentucky, during the autumn of 1830. After having received the degree of M. D. from this school he returned to Ohio, and was examined and licensed to practice, by a number of physicians appointed as censors by the Legislature to examine those who desired to practice medicine. His first office was opened in Bainbridge. His brother Joseph having studied dentistry with him previously, spent the winter of 1830 profitably in Vicksburg, Miss.. He induced his brother Joseph to return to that place with him the subsequent winter. The latter settled in Port Gibson and in Natchez. Thus, for several years he spent the winter in the South and the summer in the North. In 1834 Dr. Taylor decided to give up the practice of medicine and devote himself wholly to dentistry. He however, regarded his medical career as invaluable to his success in his present profession. At that time there were not more than a dozen dentists in the West, and few of these had made any reputation worth mentioning. Both cities and towns were small and could not afford a permanent location to a professional man. Indeed, ten years later, though the number of dentists had increased fourfold, yet very few had attained to eminence. After assuming the practice of dentistry alone, he continued his winter visits south until 1838, he had accumulated about six thousand dollars, which he invested in the dry goods business in Bainbridge, placing his youngest brother, Irwin, in charge of the store. His eyes threatened to fail him shortly after, and he feared he would be compelled to relinquish his profession, and selling out his store, he removed with his brother to Crawfordsville, Indiana, taking with him a stock of goods. Here he soon found himself in full practice, visiting Lafayette, Covington, and neighboring towns. In 1841, his merchandise venture proving unsuccessful, he closed up his business and again went South. His brother went to Marysville to study dentistry with another brother, Joseph, who had several years before settled there. Still longing for a permanent settlement, however, in 1842 Dr. James Taylor bought of Dr. Rostaing, in Cincinnati, his house, office, instruments, fixtures, and opened the practice of his chosen profession in this city, then numbering about 60,000 inhabitants. Meanwhile a fourth brother, Edward, had studied medicine and dentistry, and had pursued the same career of vibration between the North and South, and was engaging successfully in practice in Louisville, Kentucky. He was ere long induced to join his brother in Cincinnati, and in a few years they had built up a most flourishing and lucrative practice with a wide-spread reputation. After some years, however, Edward's health failed, and Dr. James Taylor took his place, while the former retired to Cleveland and engaged in horticultural pursuits there until his death, in 1867. The two remaining brothers extended their profession among the best families of the community, and became well known in the profession. Thus these three brothers--the younger and fourth practitioner having died early—laid a broad foundation for the rising profession of dentistry, and by their enthusiasm and labors in it, helped to give it that high professional character and standing which it has attained not only in the West, but throughout the country. While thus engaged in Cincinnati, Dr. James Taylor was invited to a chair in the Dental College in Baltimore, which had been organized by his former friend Prof. Chapin A. Harris; but feeling that a college of dental surgery should be established in the West, he declined the flattering offer. At this time it was a serious sacrifice in a business view to become a professor in a dental college. In 1844 Dr. Taylor first advocated the necessity of a dental school for Cincinnati. After a consultation upon the subject with Drs. Jesse W. Cook and Melancthan Rogers, they concluded to apply to the Legislature for a charter. After some opposition the charter was obtained, and in 1845 the college was organized, Dr. Taylor being assigned to the chair of practical dentistry and pharmacy. The Ohio College of Dental Surgery was the second of this kind in the country. After three years a new arrangement of chairs was made, and the institutes of dental science was allotted to Dr. Taylor, which he occupied for sixteen or eighteen years, when he voluntarily retired with the honors of emeritus professor. After this he delivered some lectures every session to the classes. He was a large stockholder in the college, and for many years was the President of the Board of Trustees, and conferred the degrees at the annual commencement upon the members of the graduating class.

He was elected President of the American Dental Convention, which met in Boston, in 1856. Dr. Taylor with his brothers, Drs. Edward and Joseph, were of those who organized and sustained the Mississippi Valley Dental Society, which was one of the first dental societies in this country, and accomplished more during the first twenty-five years of its existence than any other similar society during that period. In it were developed movements, modes, and persons, the influence of which have been felt wherever dental science and art exist. Dr. Taylor was, from the organization of this society to the time of his death, an active, influential, and, indeed, a leading member. His counsel was always sought in every important movement, and his suggestions and advice always carried great weight.

In 1847 the Dental Register of the West was established by this Society as its organ. Dr. Taylor was the only resident editor, consequently the editorial duties and labor devolved mainly upon him. After three years' continuance as editor for the Society, the Register was placed wholly in his hands, he assuming all responsibility for it; so ably was it conducted that it became popular with the profession, and attained a large subscription list.

He continued to edit and publish this journal for nine years, when he transferred it to other hands. Dr. Taylor was a ready writer, and during the time he was editor of the Register, his contributions to the literature of dental science and art were numerous, embracing almost every topic relating to dental practice, and, in many cases, being original and thorough, with discussion of subjects that had been but little, if at all, considered before. The value of these contributions was duly recognized in the fact that many of them were republished in the periodicals of this and other countries.

After he ceased his editorial work, he was not so constant in the use of his pen, but he afterwards wrote many articles for the press. His lectures were usually prepared with great care, being mainly written out in full. His addresses, also, were written out in full.

In October, 1878, he again took up the active work in the college, having been appointed Professor of Operative Dentistry. He continued to occupy that position till his death, during which time he delivered three courses of lectures. In this capacity he entered into work with all the enthusiasm of his younger days, and his ripe experience enabled him to do fully as efficient work.

That which he did in the establishment and management of the Ohio College of Dental Surgery, and his editorial labors, constitute but a part of his work for the profession. His great interest in young men preparing for the work of the profession was shown, not only in his college work, but in his ever-ready disposition to advise with and counsel students and young practitioners. For many years he had constantly under his private instruction from one to three students, over whose progress he watched with almost paternal care. The writer of these lines well remembers, in the early days of his professional career, of receiving from Dr. Taylor advice and encouragement that has not only never been forgotten, but has been cherished as amongst the pleasantest memories of a busy professional life.

Dr. Taylor did much to promote the success of professional societies; and, indeed, whatever tended to the development and upbuilding of the profession, had his earnest and hearty support.

His entire professional career was one of great interest; he entered the profession while it was yet in its infancy, especially in the West, and thus was one of the pioneers. But he lived to see it grow to a full and strong manhood, and in this work he had the gratification of knowing that he had an active part.

In 1838 Dr. Taylor married Miss R. M. Applegate, of Monongahela City, Pa., a most estimable lady. This happy union was severed by her death in 1858.

He was, in 1860, married to Miss Belle P. McMaster, of Cincinnati, an accomplished lady, beloved by all who knew her. She died in 1873.

In May, 1876, he was again married to Miss Susan A. Rogers, of Sandy Hill, N. Y., who survives him.

Dr. Taylor possessed the qualities of the Christian gentleman; affable, genial, and pleasant in his intercourse with all, and especially so with his professional brethren.

He was a man of strong constitution, and rarely ever feeble or sick except by overwork. He was a hard worker, and often did not take recreation when he much needed it. He continued in practice up to within a day or two of his death, which occurred June 12, 1881, the immediate cause of which was paralysis of the heart, induced, probably, by prostration from overwork.

Thus his life stands as a noble example of self-sacrificing work, in behalf of an honorable and useful profession.

## Figures and Tables

**Figure f1:**